# Pathogenicity and molecular characteristics of fowl adenovirus serotype 4 with moderate virulence in Guangxi Province, China

**DOI:** 10.3389/fvets.2023.1190126

**Published:** 2023-05-05

**Authors:** You Wei, Zhixun Xie, Qing Fan, Zhiqin Xie, Xianwen Deng, Sisi Luo, Xiaofeng Li, Yanfang Zhang, Tingting Zeng, Jiaoling Huang, Zhihua Ruan, Sheng Wang

**Affiliations:** Guangxi Key Laboratory of Veterinary Biotechnology, Key Laboratory of China (Guangxi)-ASEAN Cross-Border Animal Disease Prevention and Control, Ministry of Agriculture and Rural Affairs of China, Guangxi Veterinary Research Institute, Nanning, Guangxi, China

**Keywords:** fowl adenovirus serotype 4, pathogenicity, molecular characterization, medium virulent, convalescence

## Abstract

The GX2020-019 strain of fowl adenovirus serotype 4 (FAdV-4) was isolated from the liver of chickens with hydropericardium hepatitis syndrome in Guangxi Province, China, and was purified by plaque assay three times. Pathogenicity studies showed that GX2020-019 can cause typical FAdV-4 pathology, such as hydropericardium syndrome and liver yellowing and swelling. Four-week-old specific pathogen-free (SPF) chickens inoculated with the virus at doses of 10^3^ median tissue culture infectious dose (TCID_50_), 10^4^ TCID_50_, 10^5^ TCID_50_, 10^6^ TCID_50_, and 10^7^ TCID_50_ had mortality rates of 0, 20, 60, 100, and 100%, respectively, which were lower than those of chickens inoculated with other highly pathogenic Chinese isolates, indicating that GX2020-019 is a moderately virulent strain. Persistent shedding occurred through the oral and cloacal routes for up to 35 days postinfection. The viral infection caused severe pathological damage to the liver, kidney, lung, bursa of Fabricius, thymus, and spleen. The damage to the liver and immune organs could not be fully restored 21 days after infection, which continued to affect the immune function of chickens. Whole genome analysis indicated that the strain belonged to the FAdV-C group, serotype 4, and had 99.7–100% homology with recent FAdV-4 strains isolated from China. However, the amino acid sequences encoded by ORF30 and ORF49 are identical to the sequences found in nonpathogenic strains, and none of the 32 amino acid mutation sites that appeared in other Chinese isolates were found. Our research expands understanding of the pathogenicity of FAdV-4 and provides a reference for further studies.

## 1. Introduction

Fowl adenovirus belongs to the family Adenoviridae and the genus Aviadenovirus. It is a nonenveloped linear double-stranded DNA virus with a genome length of 25–46 kb that can be divided into five species, FAdV-A, B, C, D, E, and 12 serotypes (FAdV-1 to 8a and FAdV-8 to 11) (1, 2). Hexon, penton, fiber-1, and fiber-2 are the main structural proteins of the envelope of FAdV-4. The nucleocapsid proteins are primarily pX, pV, pVII, and pVIII, while nonstructural proteins include E1A, E1B, E3, E4, 100 k, and 52/55 k, among others (3). Recent research has shown that the hexon protein has the function of neutralizing antigenic sites, contains type-specific antigenic determinant clusters, and can be used for serum typing (3). The penton protein plays a crucial role in virus entry into cells. The fiber-1 protein directly triggers infections by pathogenic FAdV-4 through an axial handle-like structure (4). The region at the top of the Fiber-2 protein can bind with host cells, and the strength of binding can be used as a basis for determining the virulence of the virus. Furthermore, the fiber-2 protein has good antigenicity, can induce the production of neutralizing antibodies, and can effectively limit FAdV-4 infection (5).

Hydropericardium hepatitis syndrome (HHS) is mainly caused by fowl adenovirus serotype 4 (FAdV-4) and has previously been observed in regions such as Pakistan, Kuwait, Australia, Iraq, South America, Central America, India, Japan, and Korea (6–8), causing considerable economic losses to the global poultry industry. The virus mainly infects broiler chickens between the ages of 3–6 weeks and has a death rate of up to 80% or more in chicks (9). However, cases of infection in breeders between 12 and 25 weeks have also been reported in recent years (10). Poultry begin to show symptoms of FAdV-4 infection from the 3rd to 4th day, with a peak in the number of deaths occurring on the 5th to 6th day, followed by a decline (11). The main gross changes in diseased chickens include the accumulation of a large amount of light yellow fluid in the pericardial sac and the yellowing and noticeable swelling of the liver, and sometimes hemorrhage and necrosis can be observed on the surface of the liver (12). Previously, FAdV infections in China were primarily asymptomatic secondary infections. The first case of highly pathogenic FAdV-4 in China was reported in June 2015, and the virus then rapidly spread across the country (13). Outbreaks have occurred in major poultry-rearing provinces, such as Shandong, Henan, Jiangsu, Anhui, Sichuan, Hunan, Guangxi, Guangdong, and others (14–16). Since 2016, there have been suspected cases of HHS in cities, such as Nanning, Yulin, Guilin, and Qinzhou in Guangxi Province, seriously threatening the local poultry industry (17, 18).

In this study, an FAdV-4 strain was isolated from chickens suspected to have HHS in Guangxi Province, China, and subjected to whole-genome sequencing. It was compared with pathogenic and nonpathogenic strains published in GenBank from different regions. The effects of different inoculation doses on viral pathogenicity in chickens and after disease progression were also determined. This study aimed to provide evidence to support the prevalence of FAdV-4 in the Guangxi region and lay the foundation for the research and control of FAdV-4.

## 2. Materials and methods

### 2.1. Experimental animal and ethics statement

Specific pathogen-free (SPF) White Leghorn chicken eggs were purchased from Beijing Boehringer Ingelheim Vital Biotechnology Co., Ltd. (Beijing, China). The eggs were incubated for 20–21 days until hatching in an incubator, and the chickens were then raised in a negative pressure SPF isolation unit until 4 weeks of age to test the pathogenicity of the FAdV-4 strain. The animal experiment was approved by the Ethics Committee of Guangxi Veterinary Research Institute. The experimental procedures were conducted in accordance with the regulations of the Animal Ethics Committee of Guangxi Veterinary Research Institute (No. 2019c0406).

### 2.2. Sample collection and FAdV-4 PCR detection

In 2020, a commercial broiler flock consisting of approximately 4,000 8-week-old chickens at a poultry farm in Yulin City, Guangxi Province, South China, exhibited signs of poor health, such as reduced appetite and huddling in corners. The flock experienced a daily mortality rate of approximately 40–60 chickens, accounting for approximately 1% of the total population. Upon autopsy, typical HHS lesions were observed. The virus was isolated from liver tissue specimens showed hepatomegaly, yellow discolouration, and haemorrhagic necrosis. The samples were homogenized in phosphate-buffered saline (PBS), and after three freeze–thaw cycles, the homogenate was centrifuged at 12,000 *g* for 10 min. The supernatant was used for FAdV-4 PCR detection and virus isolation. A commercial TransGen Biotech EasyPure Genomic DNA/RNA Kit (TransGen, China) was used to extract DNA/RNA from the supernatants.

To detect the presence of viral DNA in the samples, PCR was performed using specific primers for the hexon gene of FAdV-4. The forwards primer sequence was 5’-CGAGGTCTATACCAACA CGAGCA-3′, and the reverse primer sequence was 5’-TACAG CAGGTTAATGAAGTTATC-3′. The PCR amplification protocol consisted of an initial denaturation step at 95°C for 5 min, followed by 30 cycles of denaturation at 95°C for 30 s, annealing at 56°C for 30 s, extension at 72°C for 30 s, and a final extension step at 72°C for 10 min. To isolate the virus from positive samples, the supernatants were filtered through a 0.22 μm PES membrane filter unit and inoculated into primary cultures of chicken embryo liver (CEL) cells. The culture supernatant was harvested 120 h after virus inoculation or when the cell cytopathic effect reached 7% or more. The harvested supernatant was blindly passaged three times using primary CEL cells.

### 2.3. Virus purification and identification

After the virus was isolated, dilutions of the virus were prepared for plaque purification in CEL cells. Dilutions ranging from 10^−3^ to 10^−8^ were inoculated into CEL cells in six-well cell culture plates. After 1 h of infection, the cells were treated with Dulbecco’s Modified Eagle Media/Nutrient Mixture F-12 (DMEM/F12; Gibco, United States) containing 1% low melting point agarose (Promega, United States) and 2% fetal bovine serum (Gibco, United States) and incubated for 5–6 days at 37°C and 5% CO_2_. Isolated plaques were then selected and transferred to a new fresh culture of CEL cells. Plaque purifications were performed three times. The purified isolates were named GX2020-019, propagated into seed batches, and stored at −80°C.

The identification of FAdV-4 and determination of its median tissue culture infectious dose (TCID_50_) were performed using an indirect immunofluorescent assay (IFA) to detect virus-infected cells with anti-FAdV-4 monoclonal antibodies prepared in the laboratory (19). The possibility of contamination was ruled out by PCR detection of avian influenza virus (AIV), Newcastle disease virus (NDV), infectious bronchitis virus (IBV), laryngotracheitis virus (LTV), infectious bursal disease virus (IBDV), reovirus (REV), avian leukosis virus (ALV), reticuloendotheliosis virus (REV), and Mycoplasma (20–26).

### 2.4. Full-length PCR amplification and sequencing

Thirty-nine pairs of primers were synthesized to amplify the full-length DNA segments covering the viral genome of FAdV-4 strain GX2019-010 (27). PCR was performed using PrimeSTAR HS DNA Polymerase (TaKaRa, Japan). Each 50 μL reaction contained 10 μL 5× PrimeSTAR Buffer, 4 μL dNTP mixture, 0.5 μL polymerase (2.5 U/μL), 2 μL total DNA from FAdV-4 isolates, 1 μL of each primer (10 μmol/L), and nuclease-free water to reach a final volume of 50 μL. The PCR included an initial denaturation at 98°C for 5 min, followed by 30 cycles of 10 s at 98°C, 5 s at 55°C, and 2 min at 72°C and a final extension step for 10 min at 72°C. PCR products were analyzed by 1% agarose gel electrophoresis and visualized by GelRed staining. The PCR products were sequenced directly or cloned into the pMD18-T vector for sequencing. The Seqman program, which is part of the Lasergene software package (version 7.1, DNASTAR, United States), was used to manually assemble the complete sequence.

### 2.5. Phylogenetic analysis and molecular characterization of FAdV-4

The complete nucleotide sequence of GX2020-019 was aligned with 49 reference strains of FAdV-A to E available from the GenBank database using the ClustalW multiple alignment algorithm (shown in Table 1). A phylogenetic tree was created by neighbor-joining analysis with 500 replicates for bootstrapping, and evolutionary distances were calculated using the maximum composite likelihood method through MEGA 11 software (version 11, Molecular Evolutionary Genetic Analysis, New Zealand). MegAlign software (version 7.1, DNASTAR, United States) was utilized for a full-genome similarity comparison of GX2020-019 with pathogenic strains (GX2019-004, SD1601, JS07, SCDY, SD1511, and HLJFAd15 strains), nonpathogenic strains (ON1, KR5, and B1-7 strains) of FAdV-C, and reference strains of FAdV-A, B, D, and E. The major structural protein genes and ORFs of GX2020-019 were translated into amino acid sequences using EditSeq software (version 7.1, DNASTAR, United States). These sequences were compared with the sequences of FAdV-4 pathogenic strains (MX-SHP95, GX2019-004, SD1601, JS07, SCDY, SD1511, and HLJFAd15 strains) and nonpathogenic strains (ON1, KR5, and B1-7 strains) using MegAlign software (version 7.1, DNASTAR, United States).

**Table 1:** Information of fowl adenovirus reference strains.

Strains	GenBank accession No.	Species	Serotypes	Country
HLJFAd15	KU991797.1	C	4	China
CH/SXCZ/2015	KU558762.1	C	4	China
HB1510	KU587519.1	C	4	China
JSJ13	KM096544.1	C	4	China
NIVD2	MG547384	C	4	China
AH-F19	MN781666	C	4	China
CH/AHMG/2018	MN606303.1	C	4	China
D1910497	MW711380.1	C	4	China
GD616	MW509553.1	C	4	China
JS07	KY436519	C	4	China
AQ	KY436520	C	4	China
HN	KY379035	C	4	China
AH712	KY436522	C	4	China
SCDY	MK629523	C	4	China
SD1601	MH006602	C	4	China
GX2017-004	MN577980	C	4	China
GX2019-005	MN577981	C	4	China
GX2019-010	MW439040	C	4	China
SD1511	MF496037	C	4	China
GX2019-011	MW439041	C	4	China
GX2017-001	MN577977	C	4	China
GX2018-007	MN577983	C	4	China
GX2018-008	MN577984	C	4	China
D1910497	MW711380	C	4	USA
MX-SHP95	KP295475	C	4	Mexico
ON1	GU188428.1	C	4	Canada
KR5	HE608152.1	C	4	Austria
B1-7	KU342001.1	C	4	India
CELO	U46933.1	A	1	Austria
61/11z	KX247012.1	A	1	Poland
JM1/1	MF168407.1	A	1	Japan
11–7,127	MK572848.1	A	1	Japan
OTE	MK572847.1	A	1	Japan
W-15	KX247011.1	A	1	Poland
340	NC021211.1	B	5	Ireland
WHRS	OM836676.1	B	5	China
19/7209	OK283055.1	B	5	Hungary
LYG	MK757473.1	B	5	China
AF083975	AF083975.2	D	9	Canada
SR48	KT862806.1	D	2	Austria
ON-NP2	KP231537.1	D	11	Canada
MX95-S11	KU746335.1	D	11	Mexico
380	KT862812.1	D	11	Britain
685	KT862805.1	D	2	Britain
HG	GU734104.1	E	8	Canada
UPM04217	KU517714.1	E	8b	Malaysia
764	KT862811.1	E	8b	Britain
TR59	KT862810.1	E	8a	Japan
YR36	KT862809.1	E	7	Japan

### 2.6. Pathogenicity assessment of GX2020-019

Four-week-old SPF chickens were used to evaluate the pathogenicity of GX2020-019. The chickens were divided into six groups of 10 birds in each group based on the inoculated dose of GX2020-019 (10^3^ TCID_50_, 10^4^ TCID_50_, 10^5^ TCID_50_, 10^6^ TCID_50_, and 10^7^ TCID_50_) and were inoculated *via* intramuscular injection. Control birds received an equal volume of PBS. Each group was housed separately in a negative-pressure SPF chicken isolator. The birds were monitored four times daily for 21 days and scored for clinical signs and mortality. The scale used was as follows: 0 for normal; 1 for precursory symptoms, such as depression and disorganized feathers; 2 for obvious symptoms, such as yellow and green excrement, assumption of the fetal position, and anorexia; and 3 for death. The survival rate curve and clinical signs score curve were plotted using Prism 8.0 software (GraphPad Software Inc., United States).

To assess the impact of GX2020-019 on the body, 30 four-week-old SPF chickens were injected with 10^4^ TCID_50_ *via* intramuscular injection, and another five chickens were injected with the same volume of PBS as a control group. Three chickens exhibiting obvious clinical signs, such as green feces, listlessness, weakness, prostration, and decreased appetite, were euthanized on day 5 postinoculation (dpi) and were regarded as the symptomatic group. Three chickens that had recovered from obvious clinical symptoms were euthanized and regarded as the convalescent group at 21 dpi. Additionally, three chickens from the PBS control group were euthanized. Gross lesions were examined and observed. Tissue samples were collected from the liver, heart, spleen, glandular stomach, pancreas, lung, kidney, bursa of Fabricius, thymus, brain, muscle, small intestine, muscular stomach, esophagus, and trachea. A portion of each tissue sample was fixed in 10% neutral formalin buffer solution for histological analysis, while another portion was stored at −80°C for viral load detection in the tissues.

### 2.7. Detection of viral shedding

To determine the duration and concentration of viral shedding in the infected chickens, 20 4-week-old SPF chickens were intramuscularly injected with 10^3^ TCID_50_ of GX2020-019, while another five chickens were used as a control group and received the same volume of PBS. Oral and cloacal swabs were collected from 10 birds at 1, 3, 5, 7, 10, 14, 21, 28, and 35 dpi to extract viral DNA and detect FAdV-4 viral loads.

### 2.8. Examination of histopathology

The tissue samples were fixed in a 10% neutral formalin buffer solution at room temperature for at least 48 h and then sent to Guangzhou Maike Biotechnology Co., Ltd. (Guangzhou, China) for tissue sectioning and hematoxylin–eosin (H&E) staining. Lesions associated with FAdV-4 infection were observed under an electron microscope and photographed.

### 2.9. Detection of viral loads

A total of 0.2 g of each tissue sample was homogenized in 1 mL of PBS. The homogenate was subjected to three freeze–thaw cycles, followed by centrifugation at 12,000 *g* for 10 min. Then, 300 μL of supernatant was collected for DNA extraction using a Universal Genomic DNA Kit (CoWin Biotech Co., Ltd., China) according to the manufacturer’s instructions. The concentration of the extracted nucleic acid was determined using a NanoDrop ND1000 spectrophotometer (Thermo Scientific, United States) and standardized to 100 μg/mL for use as a template in real-time PCR. Oral and cloacal swabs were suspended in 1 mL of PBS and agitated for 30 s. The suspension was then allowed to settle at room temperature, and 300 μL of the resulting supernatant was used for DNA extraction with a real-time PCR kit.

All samples were tested in triplicate using the following detection primers: 5′GCACGAGG CACCTCCAAAGAG3′ and 5′GTTGTACCC GTCGCAG GAGGATG3′. Real-time PCR was conducted using a 20 μL total volume containing 2 μL of template DNA, 10 μL of PowerUp TM SYBRTM GreenMasterMix, 1 μL of each primer (10 μmol/L), and 10 μL of deionized water. The thermal cycling program included an initial cycle at 95°C for 120 s, followed by 40 cycles of denaturation at 95°C for 15 s and annealing/extension at 60°C for 60 s (28). The melting curve was analyzed at the end of the cycling program. Viral load was calculated based on the Ct value of the sample and the standard curve. All samples were analyzed in triplicate.

### 2.10. Statistical analysis

Statistical analysis was performed using one-way ANOVA with Prism 8.0 (GraphPad Software Inc., United States). Results with a *p* value of less than 0.05 were considered statistically significant. The results are presented as the means ± SDs.

## 3. Results

### 3.1. FAdV-4 isolated from chickens with HHS

After DNA extraction from the liver tissue homogenate, a positive band was observed by PCR using the FAdV-4,421 bp detection primer. The supernatant of the homogenate was then inoculated into CEL cells, and significant cytopathic effects were observed in the first generation after inoculation as the cells became round. The cytopathic effect in the second and third generations reached 70% and remained stable at approximately 72 h, indicating good viral replication in CEL cells. The FAdV-4 isolate was purified by three rounds of plaque purification and named GX2020-019.

An IFA was performed and showed that GX2020-019 infected cells had a significant positive reaction with the anti-fiber-2 monoclonal antibody against FAdV-4. Contamination by AIV, NDV, IBV, LTV, IBDV, RLV, ALV, REV, and Mycoplasma in the viral liquid was ruled out by PCR testing. The TCID_50_ of the seed virus stock was determined to be 10^8.2^/0.1 mL.

### 3.2. Pathogenicity of the virus in SPF chickens infected with different doses of GX2020-019

**Figure 1:**
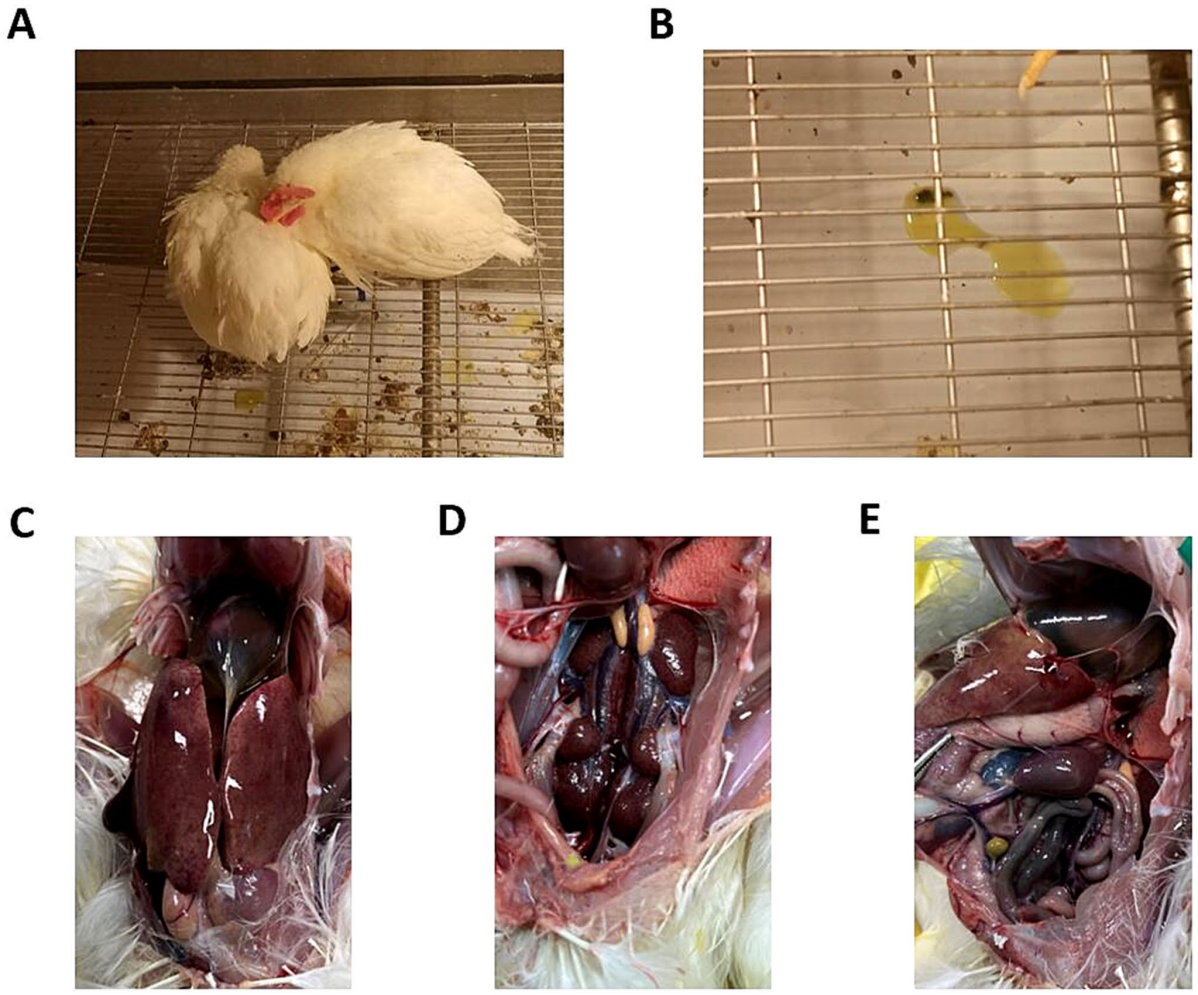
Clinical symptoms and gross lesions in chickens infected with GX2020-019. **(A)** Depressed mental state, fluffed feathers, anorexia, and somnolence in the diseased chickens. **(B)** Yellow-green watery stools. **(C)** Presence of effusion in the pericardium, enlarged liver, liver yellowing, and bleeding spots. **(D)** Congested and enlarged kidneys. **(E)** Congested and enlarged spleen.

**Figure 2:**
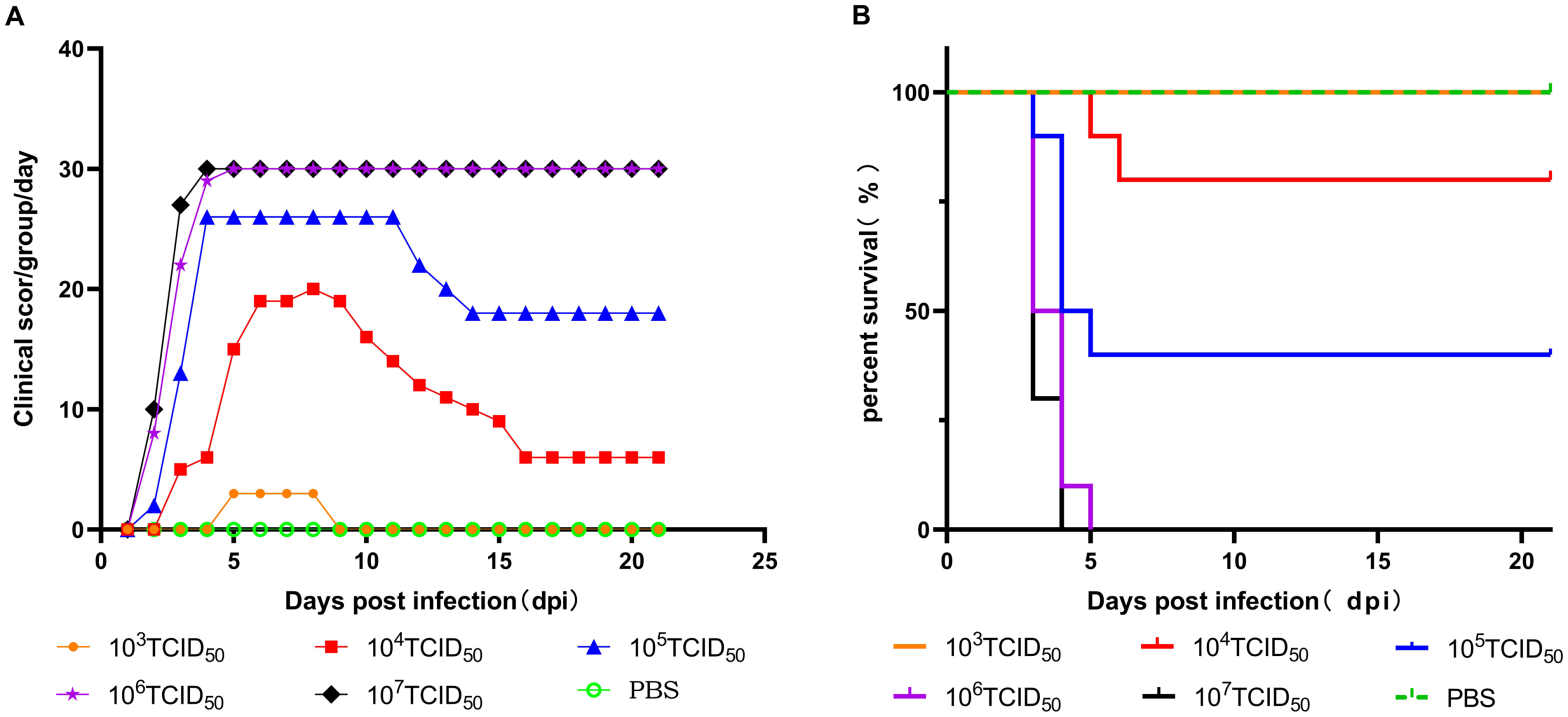
Pathogenicity of the GX2020-019 strain in SPF chickens at different doses. **(A)** Clinical symptom scores of SPF chickens inoculated with different doses of the GX2020-019 strain. **(B)** Survival curve of SPF chickens inoculated with different doses of the GX2020-019 strain.

Five different doses were used to infect chickens through the intramuscular route. After 48 h of infection, chickens in the 10^6^ TCID_50_ and 10^7^ TCID_50_ groups showed clinical symptoms of depression, fluffy feathers, and yellow-green soft stools. At 4 dpi, 100% of the chickens died, and necropsy revealed large amounts of light-yellow effusion in the pericardium, yellowing and fragile liver with obvious blood spots on the surface, as well as congested and enlarged kidneys and spleens (shown in Figure 1). The average time to death was 79 h in the 10^6^ TCID_50_ group and 70 h in the 10^7^ TCID_50_ group. The time at which the number of deaths peaked in the 10^4^ TCID_50_ and 10^5^ TCID_50_ groups was 4–6 dpi, with a morbidity rate of 90 and 100% and a mortality rate of 20 and 60%, respectively. The average time of death was 86 and 124 h in the two groups, respectively. The symptoms of the chickens that did not die were significantly reduced 6–8 days after onset, appetite returned, and the chickens began to recover. No obvious clinical symptoms or death occurred in the 10^3^ TCID_50_ group, and only 30% of chickens showed mental depression from 5 to 8 dpi, which recovered to the levels in the control group. No birds in the control group became sick or died. The clinical symptom score and the survival curve are shown in Figure 2.

### 3.3. Histopathological changes in SPF chickens infected with GX2020-019

During the disease period of chickens infected with GX2020-019, mild to severe degenerative necrosis and widespread inflammatory cell infiltration were found in the liver, bursa of Fabricius, spleen, thymus, lung, and kidney. In particular, inclusion bodies formed within the liver cells, and the lung alveolar cavities were filled with shedding dead cells, red blood cells, and inflammatory cells.

**Figure 3:**
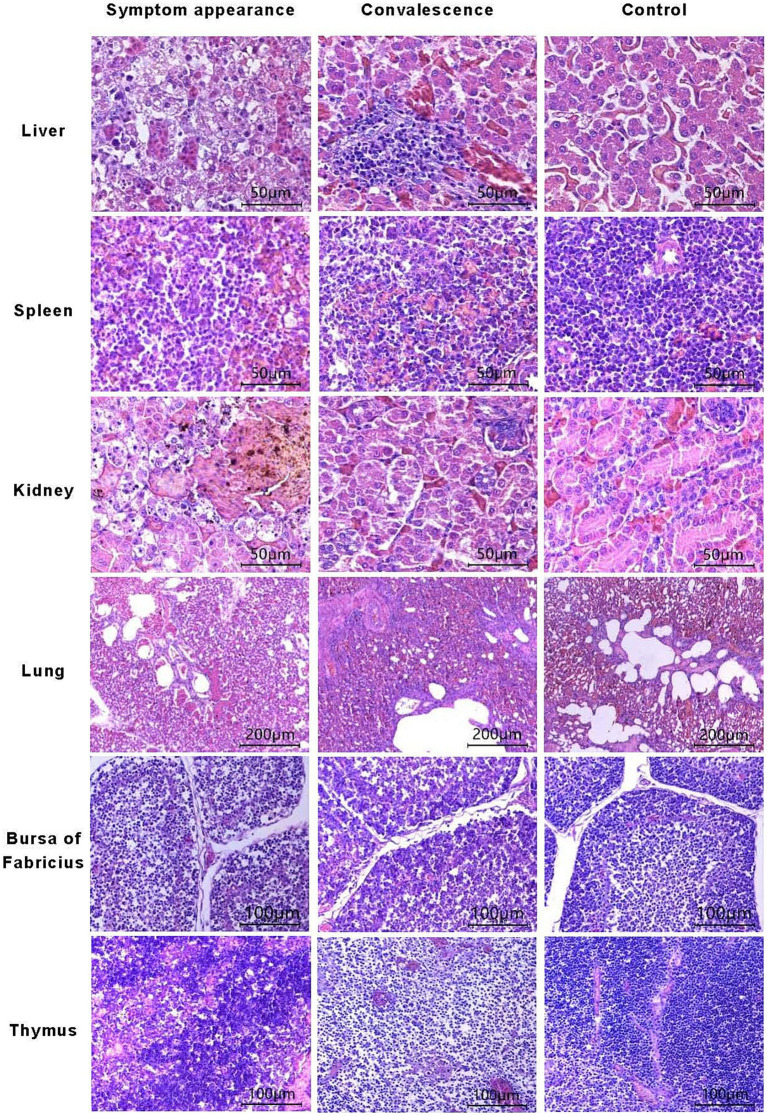
Pathological changes in organ tissues during onset and convalescence.

During the convalescence period, the level of liver cord degeneration was reduced, but there was still monocyte infiltration around the hepatic sinusoids, which remained narrow. The congestion and stasis in the lung disappeared, and the exudate in the lung alveoli was absorbed, while the lung alveolar wall cells were the same as those in the control group. The level of atrophy of the lymph follicles in the bursa of Fabricius was reduced, and the distinction between the medulla-cortex boundary and the interfollicular septa became clearer. The degree of necrosis in the thymus cells was reduced, the ratio of cortex to medulla increased, the level of apoptotic lymphocytes decreased, and the abundance of medullary blood vessels and thymic corpuscles increased. Interstitial hemorrhages in the spleen tissue disappeared, and the state of the lymphocytes changed from degenerative necrosis to mild degeneration, with clear distinction in the germinal center. The state of the kidney changed from exhibiting focal necrosis of renal tubular epithelial cells to exhibiting necrosis of individual renal tubular epithelial cells (shown in Figure 3). The heart, glandular stomach, pancreas, brain, muscle, small intestine, fundus, esophagus, and trachea remained the same as those in the control group during the observation period, with no significant pathological changes.

### 3.4. The viral load of various organs in chickens infected with GX2020-019

**Figure 4:**
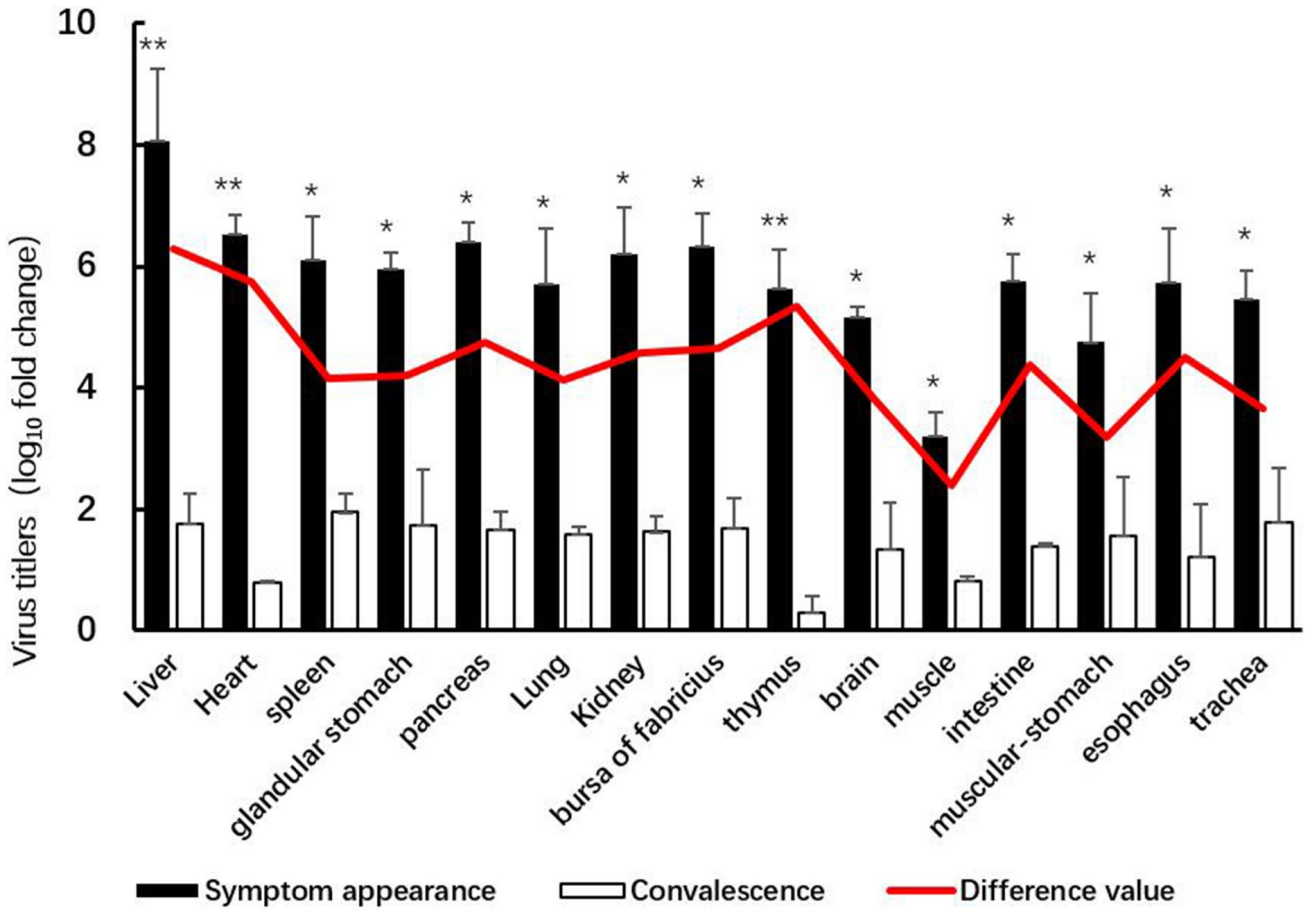
Organ viral loads in SPF chickens infected with GX2020-019.

The GX2020-019 strain of FAdV-4 is widely tissue tropic in chickens, with high concentrations of the virus detected in all organs and the highest viral load in liver tissue, which is the main target organ; the muscle had the lowest viral load. After entering the regression period, the viral content of each organ significantly decreased (*p* < 0.05), with the thymus and heart having the lowest viral loads and the liver, heart, and thymus showing the highest efficiency of viral clearance (shown in Figure 4).

### 3.5. Viral shedding in SPF chickens after GX2020-019 infection

**Figure 5:**
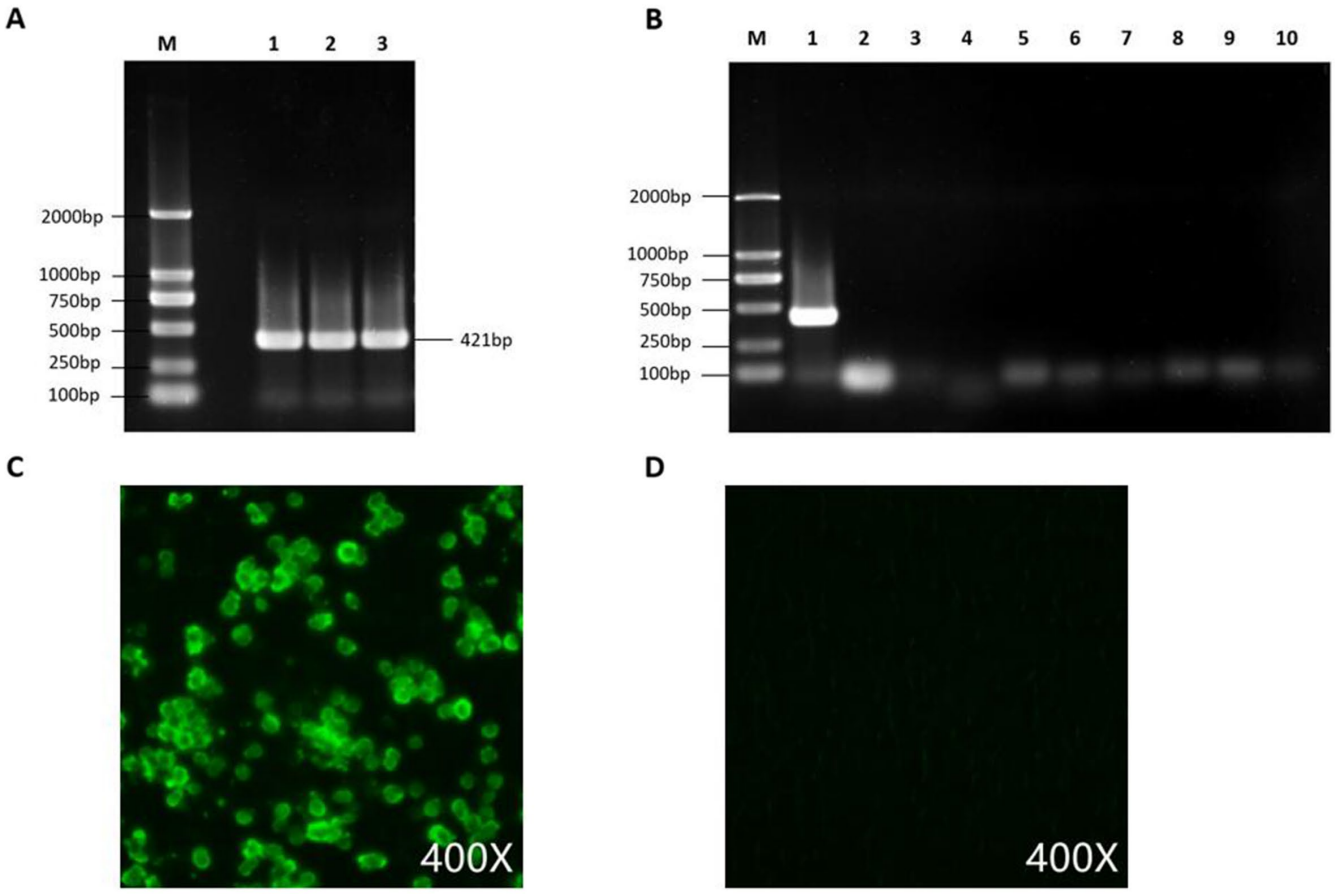
Detection and purity testing of FAdV-4 virus. **(A)** FAdV-4 virus was detected using conventional PCR. **(B)** PCR was used to rule out contamination with common mixed infection pathogens, with lanes 1–10 showing detection results for FAdV-4, AIV, NDV, IBV, LTV, IBDV, RLV, ALV, REV, and Mycoplasma. **(C)** GX2020-019 was identified using an anti-FAdV-4 fiber-2 monoclonal antibody by IFA. **(D)** Negative control.

**Figure 6:**
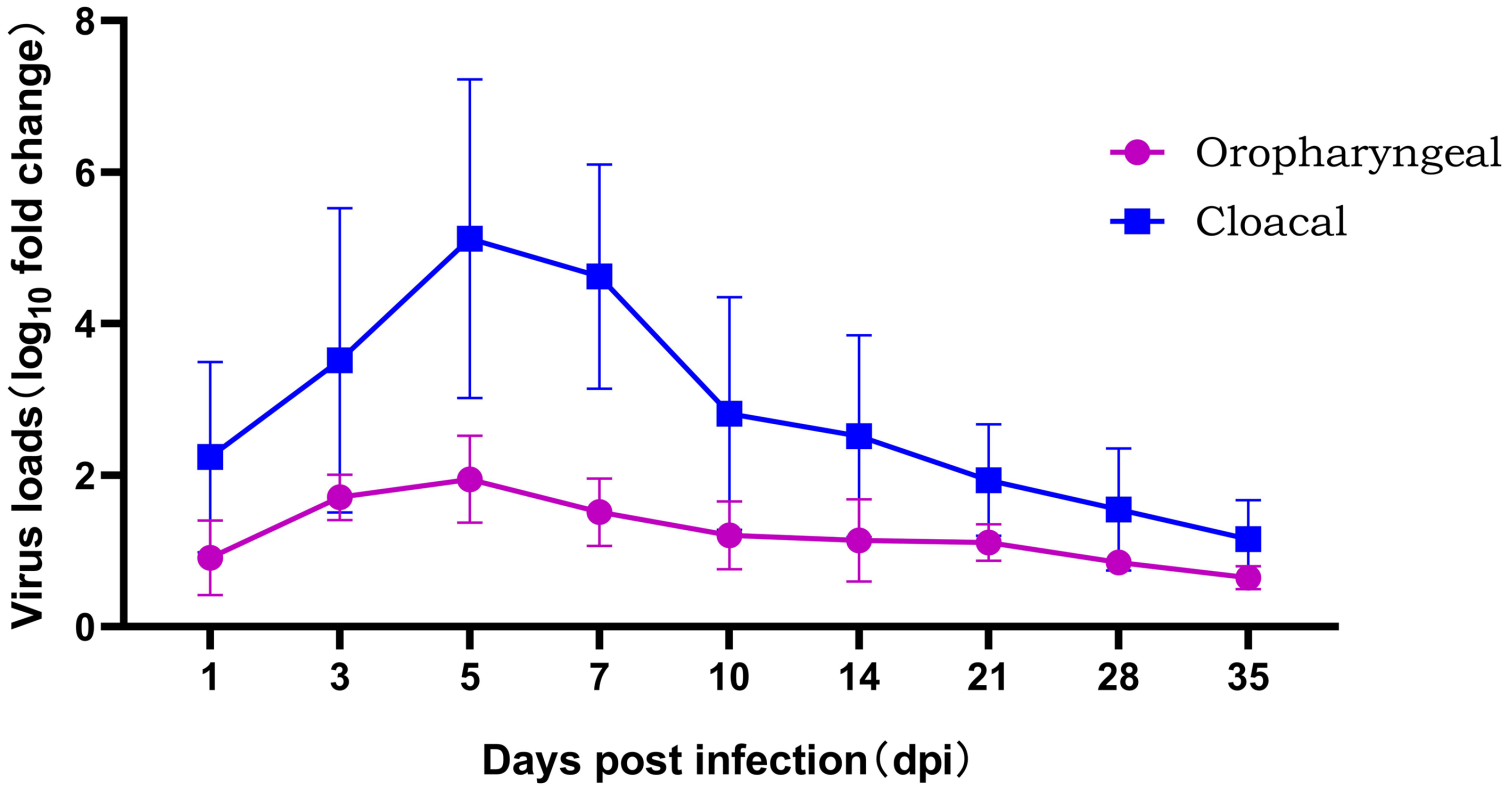
Viral shedding in SPF chickens infected with GX2020-019.

The level of viral shedding in the oropharyngeal and cloaca was quantified by fluorescence quantitative PCR, as shown in Figure 5. From 1 to 35 dpi, the level of viral shedding in infected chickens showed a trend of first increasing and then decreasing. The peak level of viral shedding in the cloaca occurred between 5 and 7 dpi, at which time the level of shedding was significantly higher than that in the oropharyngeal area (*p* < 0.01). A small amount of viral shedding was still detectable in both the oral cavity and cloaca at 35 dpi (shown in Figure 6). The results of FAdV-4 shedding detection in the control group were always negative.

### 3.6. Sequence alignment and phylogenetic analysis

**Figure 7:**
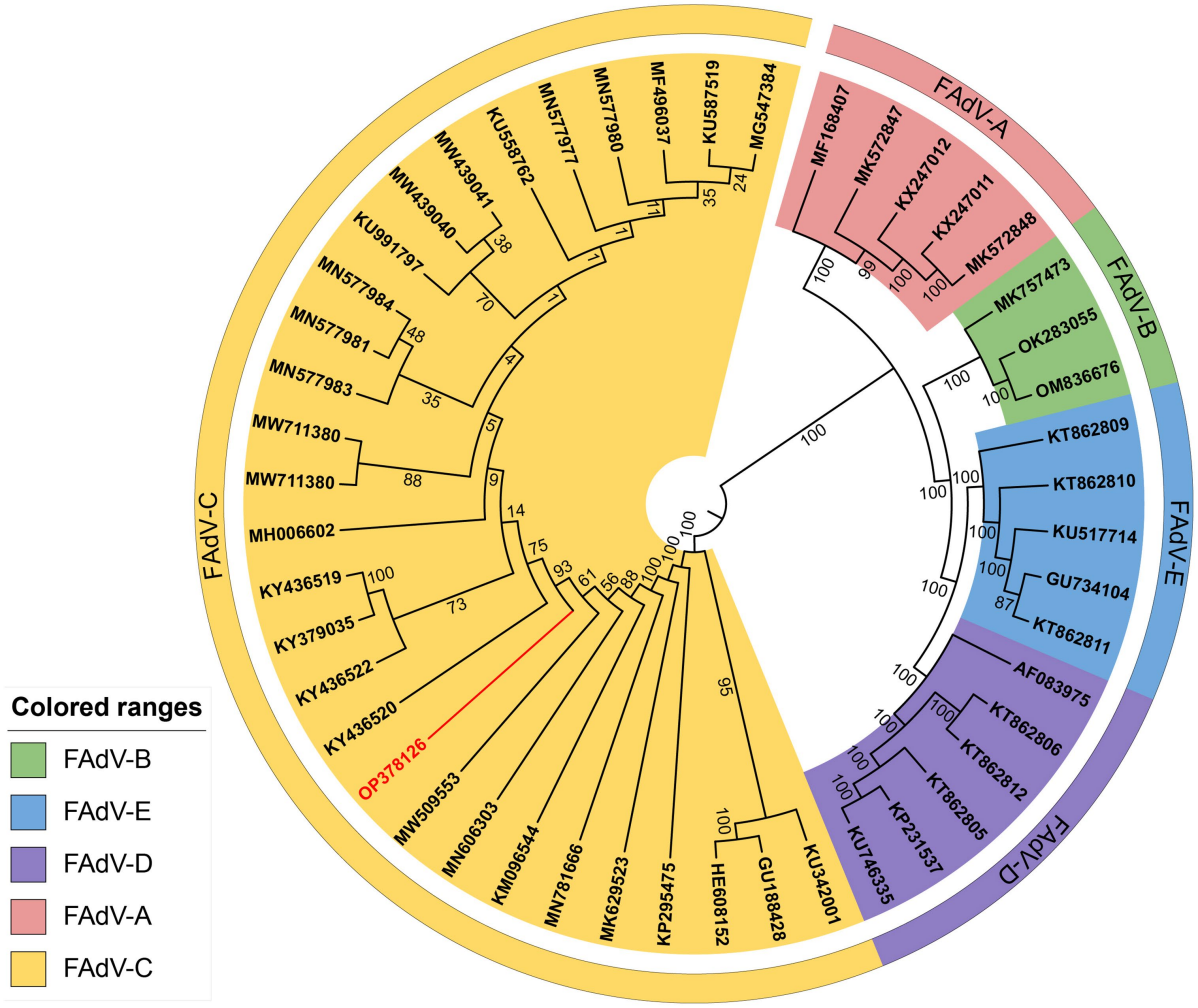
Phylogenetic analysis of the whole gene nucleotide sequence of strain GX2020-019 of Guangxi. The phylogenetic tree was created by neighbor-joining analysis with 500 replicates for bootstrapping, and evolutionary distances were calculated using the maximum composite likelihood method through MEGA 11 software. The GX2020-019 strain is indicated in red.

**Figure 8:**
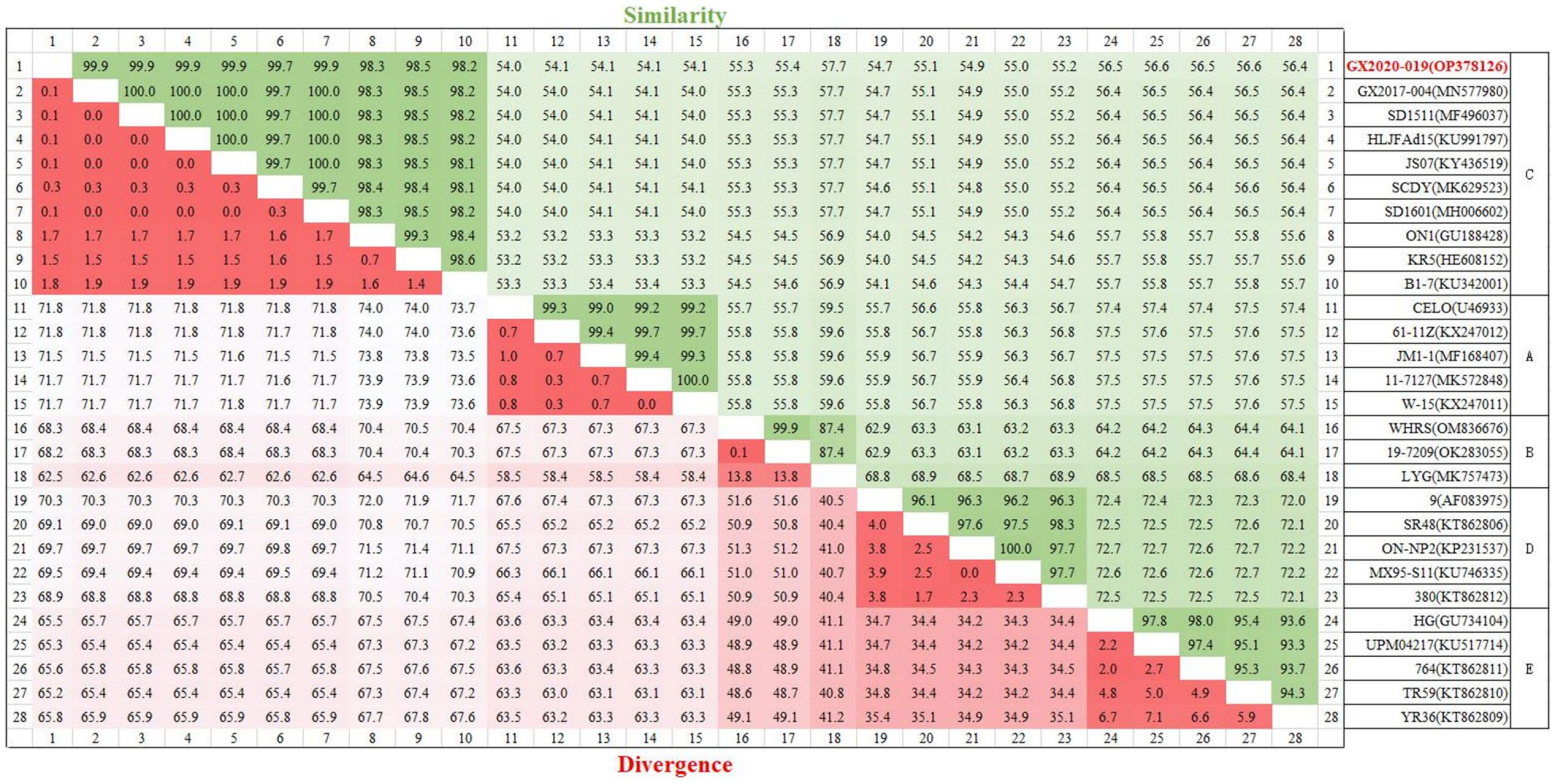
Homology analysis of the whole genomic sequence of the GX2020-019 strain. The GX2020-019 strain is indicated in red. Homology analysis between the GX2020-019 strain and reference strains using the MegAlign program within the Lasergene7.0 software package.

The genome of GX2020-019 is 43,714 nucleotides in length, with A, T, G, and C contents of 23.1, 22.0, 27.2, and 27.7%, respectively, and contains 43 potential protein-coding regions (GenBank accession number: OP378126). Phylogenetic analysis indicated that GX2020-019 belongs to the FAdV-C group along with FAdV-4 strains from China, Canada, India, and Austria (shown in Figure 7) and is distantly related to FAdV-A, FAdV-B, FAdV-D, and FAdV-E with genome homologies of 54.0–54.1%, 55.3–57.7%, 54.7–55.2%, and 56.4–56.6%, respectively. The homology between GX2020-019 and FAdV-4 strains from China (GX2017-010, SD1511, HLJFAd15, JS07, SCDY, and SD1601) ranges from 99.7 to 100%, while the homology with strains from outside China (ON1, KR5, and B1-7) ranges from 98.1 to 99.3% (shown in Figure 8).

### 3.7. The insertion/deletion nucleotide sites of GX2020-019

**Figure 9:**
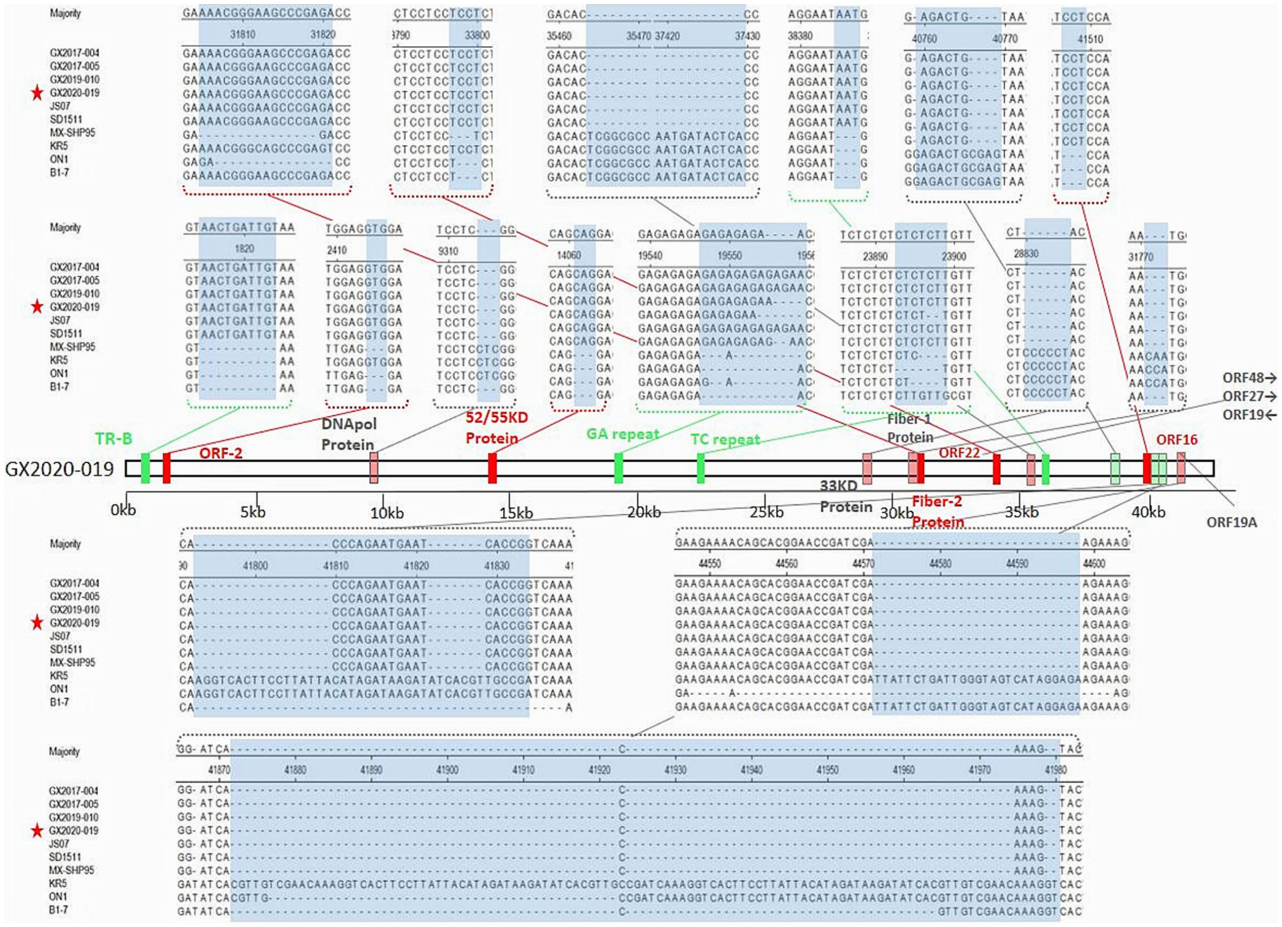
The insertion/deletion nucleotide sites of the whole genomic sequence of the GX2020-019 strain. The green region represents the inserted noncoding sequence (CDS) region. The red region represents the inserted coding sequence (CDS) region. The light green region represents the missing noncoding sequence (CDS) region. The light red region represents the missing coding sequence (CDS) region. The blue region represents the observed changes.

Unlike the oversea strains MX-SHP95, KR5, ON1, and B1-7, GX2020-019 and other FAdV-4 strains reported in China have a 10-base insertion in the tandem repeat sequence (TR-B), three-base insertions in the noncoding regions after the 52/55 kDa protein coding sequence and ORF43, a six-base deletion in the 33 kDa protein coding sequence, long GA sequences in the GA repeat region between the Px and PVI genes, and a deletion of 1,966 bases at ORF19, ORF27, and ORF48, resulting in the almost complete loss of these three ORFs. Unlike nonpathogenic strains, pathogenic FAdV-4 has a three-base insertion in ORF16, resulting in an additional glycine. In addition, unlike the ON1 strain, Chinese isolates including GX2020-019 have a three-base insertion in the coding regions of ORF2, ORF22, and DNApol protein, a 57-base insertion in ORF19A, a 15-base insertion in the coding sequence of fiber-2 resulting in an addition of 5 amino acids (ENGKP), a three-base deletion in the coding region at the end of fiber-1 resulting in a loss of a histidine residue, a five-base deletion in the noncoding region between ORF17 and ORF30, an 81-base deletion in the tandem repeat sequence (TR-E), and long TC repeat sequences in the TC repeat region between the protease and DBP genes (shown in Figure 9).

### 3.8. Protein alignment analysis of GX2020-019

The amino acid sequence lengths of the major structural proteins hexon, penton, fiber-1, and fiber-2 in the GX2020-019 strain are 937 amino acids (AA), 525 AA, 431 AA, and 479 AA, respectively. Unlike the amino acid sequences of nonpathogenic strains, they have 10–11, 6–7, 10–20, and 30–32 amino acid mutation sites, respectively. Penton and hexon are relatively conserved, while the fiber-2 protein has the most mutation sites, and the mutation positions are consistent with previous reports of other isolated strains in China (11.17.29).

Of note, we found that the amino acid sequences encoded by ORF30 and ORF49 in the GX2020-019 strain were the same as those of nonpathogenic strains, and no mutations occurred in the 32 amino acid mutation sites that were mutated in other strains isolated in China (shown in Table 2). It is inferred that there is a possibility of gene recombination between pathogenic and nonpathogenic FAdV-4 strains.

**Table 2:** Differences in the amino acid sequences of ORF30 and ORF 49 between GX2020-019 and the reference strains.

CDS	Strain	GenBank	Mutation sites													
ORF30			8	23	40	49	51	63	71							
	GX2020-019	OP378126	S	R	W	S	E	E	G							
	B1-7	KU342001.1	•	•	•	•	•	•	•							
	KR5	HE608152.1	•	•	•	•	•	•	•							
	ON1	GU188428.1	•	•	•	•	•	•	•							
	MX-SHP95	KP295475	-	Q	•	T	G	V	•							
	GX2017-004	OP378126	-	Q	-	T	G	V	R							
	SD1511	MF496037	-	Q	-	T	G	V	R	-						
	HLJFAd15	KU991797	-	Q	-	T	G	V	R	-						
	SCDY	MK629523	-	Q	-	T	G	V	R	-						
	SD1601	MH006602	-	Q	-	T	G	V	R	-						
ORF49			26	28	29	30	31	32	33	34	35	36	37	38	39	40
	GX2020-019	OP378126	D	R	S	H	D	C	Y	V	T	E	G	G	A	S
	B1-7	KU342001.1	•	•	•	•	•	•	•	•	•	•	•	•	•	•
	KR5	HE608152.1	•	•	•	•	•	•	•	•	•	•	•	•	•	•
	ON1	GU188428.1	•	•	•	•	•	•	•	•	•	•	•	•	•	•
	MX-SHP95	KP295475	T	-	V	T	-	L	L	R	Q	R	R	R	S	F
	GX2017-004	OP378126	S	-	V	T	-	L	L	R	Q	R	R	R	S	F
	SD1511	MF496037	S	-	V	T	-	L	L	R	Q	R	R	R	S	F
	HLJFAd15	KU991797	S	-	V	T	-	L	L	R	Q	R	R	R	S	F
	SCDY	MK629523	S	-	V	T	-	L	L	R	Q	R	R	R	S	F
	SD1601	MH006602	S	-	V	T	-	L	L	R	Q	R	R	R	S	F
			41	43	44	46	47	48	49	50	51	52	53			
	GX2020-019	OP378126	D	E	N	S	L	Y	Q	S	L	T	-			
	B1-7	KU342001.1	•	•	•	•	•	•	•	•	•	•	•			
	KR5	HE608152.1	•	•	•	•	•	•	•	•	•	•	•			
	ON1	GU188428.1	•	•	•	•	•	•	•	•	•	•	•			
	MX-SHP95	KP295475	G	R	K	F	A	L	S	I	I	N	L			
	GX2017-004	OP378126	G	R	K	F	A	L	S	I	I	N	L			
	SD1511	MF496037	G	R	K	F	A	L	S	I	I	N	L			
	HLJFAd15	KU991797	G	R	K	F	A	L	S	I	I	N	L			
	SCDY	MK629523	G	R	K	F	A	L	S	I	I	N	L			
	SD1601	MH006602	G	R	K	F	A	L	S	I	I	N	L			

• indicates that the amino acid at the corresponding position is the same as that in the GX2020-019 strain. — indicates a deletion of the amino acid at the corresponding position in each strain.

## 4. Discussion

Since the first report in 2015 of the pathogenic FAdV-4 strain JSJ13 isolated from chickens with HHS (13), pathogenic FAdV-4 has spread extensively in China. Prior to the widespread use of commercial fowl adenovirus vaccines, the incidence of HHS in broilers and layers increased annually, resulting in considerable economic losses and raising public awareness of the disease. Consequently, HHS and its causative pathogen FAdV-4 have become a prominent research topic among veterinary researchers in recent years.

In 2020, a strain of the FAdV-4 virus named GX2020-019 was isolated from chickens with symptoms of HHS in a commercial farm in Guangxi Province. It has been reported that most FAdVs grow well in primary chicken embryo liver cells, primary chicken embryo kidney cells, and chicken liver cancer cell lines (LMH cells) (29). As the highest viral load is observed in the liver, initially, two purification methods using LMH cells and CEL cells were used in this research. However, the growth rate of plaques formed in LMH cells was slow, and the plaque edges were unclear, making it difficult to select plaques. Therefore, CEL cells were selected for viral purification. After three consecutive rounds of plaque purification in CEL cells, a seed stock with a titer of 10^8.2^ TCID_50_/0.1 mL was obtained.

In the pathogenicity study of GX2020-019 in SPF chickens, we determined its virulence by injecting different doses of the virus into the pectoral muscles of 4-week-old SPF chickens. Each dosage group was kept separate in individual chicken isolators. The course of disease in infected chickens was affected by the inoculation dose, but they all exhibited clinical signs, such as depression, ruffled feathers, decreased appetite, and excretion of yellow–green watery feces. Upon autopsy, typical lesions of pathogenic FAdV-4 infection were observed, including 1–3 mL of bright yellow effusion in the pericardium, significant hepatomegaly with yellowing, friable texture, and bleeding points on the surface of the liver.

At present, there is no systematic virulence evaluation method for fowl adenovirus, and only a general classification of pathogenic and nonpathogenic strains based on whether they cause disease in chickens exists. Zhao et al., orally inoculated 3-week-old SPF chickens with 10^3.5^ TCID_50_ of the JSJ13 strain, resulting in a mortality rate of 28.6% (13). Luan et al., intramuscularly injected and orally administered 10^3^ TCID_50_ of the GX2019-010 strain to 4-week-old SPF chickens, resulting in a 100% mortality rate (30). Yuan et al., intramuscularly injected 10^2.5^ TCID_50_ of the GD616 strain into 3-week-old SPF chickens, resulting in a mortality rate of 100% (29). Mo et al., inoculated SPF chickens at 7, 21, and 35 days of age with 3 × 10^3.2^ TCID_50_ of the SD1511 strain through intramuscular injection, resulting in mortality rates of 93, 80, and 100%, respectively. When administered through the intranasal route, the mortality rates were 50, 57.2, and 50% for the respective age groups (11). The study showed that the GX2020-019 strain had moderate virulence, as evidenced by mortality rates of 0, 20, and 60% when intramuscularly injected at doses of 10^3^ TCID_50_, 10^4^ TCID_50_, and 10^5^ TCID_50_, respectively, in 4-week-old SPF chickens. These mortality rates were much lower than those reported for highly pathogenic isolates in China, indicating that the GX2020-019 strain should be classified as a strain with intermediate virulence.

By conducting pathological histological studies and viral load measurements in 15 organs, including the liver, heart, spleen, proventriculus, pancreas, lung, kidney, bursa of Fabricius, thymus, brain, muscle, small intestine, gizzard, esophagus, and trachea, during both the onset and recovery stages, we found that the liver is the primary target organ of GX2020-019 infection, which can cause liver damage characterized by basophilic nuclear inclusions, lipid degeneration, and multifocal necrosis. Moreover, the liver exhibited the highest viral load during the acute stage. Although the clearance rate of the virus in the liver is high, the hepatic lobular architecture remained unclear during the recovery phase, and the sinusoids were narrowed with inflammatory cell infiltration around the blood vessels. The incomplete recovery of the liver directly affects the functions of oxidative metabolism, glycogen storage, protein synthesis, and bile secretion in the body, leading to reduced weight gain and the decreased economic value of the animals. GX2020-019 displays an affinity for immune tissues that leads to significant cellular degeneration in the bursa of Fabricius, thymus, and spleen during the acute phase of infection. Even during the recovery phase, at 21 dpi, partial lymphofollicular atrophy and blurred follicular-interfollicular boundaries were observed in the bursa of Fabricius, as well as cellular necrosis, increased vascularity in the medulla, and increased size of thymic corpuscles in the thymus. The spleen exhibited only mild lymphocyte degeneration. Damage to immune organs results in immunological impairment, which may decrease the chicken population’s resistance to other pathogenic microorganisms. During the recovery phase, only sporadic individual tubular necrosis was observed in the kidneys, which did not affect their urogenital system function.

Although the accumulation of pericardial fluid is a characteristic pathology of HHS, there was no observed tissue damage or infiltration of inflammatory cells in the heart tissue, indicating that the effusion was not a result of apoptosis or inflammation of myocardial cells. These findings are consistent with the results reported by Li et al. (31), and Niu et al. (32), which confirmed that pericardial effusion is derived from vascular exudation based on measurements of components such as total protein, albumin, aspartate aminotransferase, and creatine kinase isoenzymes. Additionally, no pathological changes were observed in the digestive system during the onset period, suggesting that the virus does not cause harm to the digestive system.

Since the outbreak of pathogenic FAdV-4 in China, the factors contributing to its increased virulence have been a hot topic of research. To identify the key virulence factors, pathogenic and nonpathogenic strains were compared using nucleotide and amino acid sequence analysis, and hypothetical nucleotide fragments or amino acid sites were replaced using reverse genetics systems. Recently, studies have shown that the 1,966 bp nucleotide fragment encoding ORF19, ORF27, and ORF48, which are missing in the novel strains, are not the factors that influence virulence (33); through R188I mutation of the hexon protein, the amino acid residue at position 188 was determined to be a key residue for reducing pathogenicity (34); through CRISPR/Cas9 gene editing of fiber-1 (35, 36) and fiber-2 (37), FAdV-4 strains with highly attenuated virulence could be obtained. Additionally, there are differing views that the high pathogenicity factors of FAdV-4 are not related to fiber-2 (34, 35) and that the increased virulence of hypervirulent FAdV-4 is independent of fiber-1 and penton (38). When comparing GX2020-019 with other highly pathogenic Chinese FAdV-4 strains, including GX2019-004, SD1601, JS07, SCDY, SD1511, and HLJFAd15, we found no differences in the main structural proteins hexon, penton, fiber-1, and fiber-2. However, interestingly, we found that the amino acid sequences encoded by ORF30 and ORF49 in the GX2020-019 strain were identical to those of nonpathogenic strains, and the 32 amino acid mutation sites that were present in other isolated strains in China were not present in GX2020-019. Although there are currently no studies on the functional role of the amino acid sequences encoded by ORF30 and ORF49, it is speculated that they may be factors influencing the pathogenicity of FAdV-4.

Our work has expanded the understanding of the pathogenicity and molecular characteristics of the moderately pathogenic FAdV-4 strain GX2020-019 that is prevalent in Guangxi Province, China, and provided reference materials for further research on this virus.

## Data availability statement

The datasets presented in this study can be found in online repositories. The names of the repository/repositories and accession number(s) can be found in the article/supplementary material.

## Ethics statement

The animal study was reviewed and approved by the regulations of the Animal Ethics Committee of Guangxi Veterinary Research Institute (No. 2019c0406).

## Author contributions

ZhixX and YW conceived and designed the experiments. YW, QF, and ZhiqX performed the experiments. XD and SL analyzed the data. XL, YZ, TZ, JH, ZR, and SW contributed reagents, materials, and analysis tools. YW wrote the paper. All authors contributed to the article and approved the submitted version.

## Funding

This work was supported by the Guangxi BaGui Scholars Program Foundation (2019A50), the Guangxi Science Base and Talents Special Program (AD17195083), and the Youth Science Fund Project in Guangxi (2020GXNSFBA297104).

## Conflict of interest

The authors declare that the research was conducted in the absence of any commercial or financial relationships that could be construed as a potential conflict of interest.

## Publisher’s note

All claims expressed in this article are solely those of the authors and do not necessarily represent those of their affiliated organizations, or those of the publisher, the editors and the reviewers. Any product that may be evaluated in this article, or claim that may be made by its manufacturer, is not guaranteed or endorsed by the publisher.
